# Management of mucormycosis coexisting with aspergillosis in pediatric age group – a case report

**DOI:** 10.1186/s43163-023-00451-x

**Published:** 2023-05-22

**Authors:** Ashish Gopal, Ishwar Singh, Nikhil Arora, Ditixaben J. Patel, Pooja Nakhat Jain, Sakshi Negi, Shramana Mandal

**Affiliations:** 1 Department of ENT and Head and Neck Surgery, Maulana Azad Medical College and Lok Nayak Hospital, New Delhi, India; 2 Department of Pathology, Maulana Azad Medical College and Lok Nayak Hospital, New Delhi, India

**Keywords:** Mucormycosis, Aspergillosis, Liposomal amphotericin, Debridement

## Abstract

**Background:**

Mucormycosis is a highly infectious deadly disease if left untreated. This disease is usually seen more in people having immunocompromised conditions like diabetes mellitus, steroid use, and neutropenia. Its presence along with *Aspergillus* is quite uncommon in the pediatric age group. This report will add information regarding such coexisting fungal disease in the pediatric age group and its further management.

**Case report:**

In this study, investigators are presenting a case of mucormycosis coexisting with aspergillosis in 3-month-old male child who presented with a palatal defect. He underwent both medical management with liposomal amphotericin B and surgical debridement for necrotic foci removal.

**Conclusion:**

This case report deals with the management of coexisting mucormycosis with aspergillosis using a combination of surgical and medical management.

## Background

COVID times were the days when mucormycosis became fatal and was rapidly growing and engulfing many patients under its trap. The aftereffects of this disease were severe and debilitating. Those patients who recovered were forced to live with facial disfigurement, blindness, paraplegia, and many more serious disability throughout their remaining life. Mucormycosis with co-existing aspergillosis is extremely rare in infancy. This paper is unique as it deals with a coexisting case of mucormycosis with aspergillosis in a 3-month-old child who presented with a palatal defect. It was a challenge for us to deal with such coexisting two fatal disease in a pediatric age group. Due to the invasive nature of the disease, debridement was planned, and all the necrotic foci were removed. Voriconazole was not started in this patient which is usually given in case of aspergillus infection as it can further make mucormycosis aggressive, so the patient was started on liposomal amphotericin B.

## Case report

**Fig. 1:**
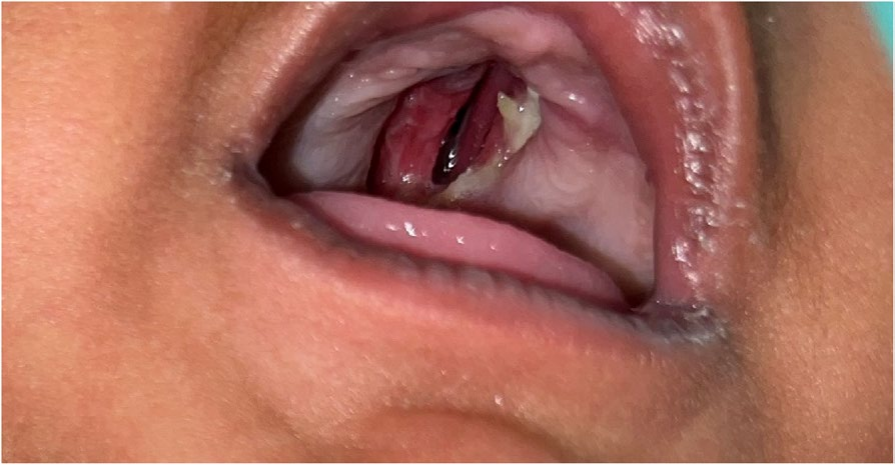
Showing palatal ulceration with discharge at the left margin

**Fig. 2:**
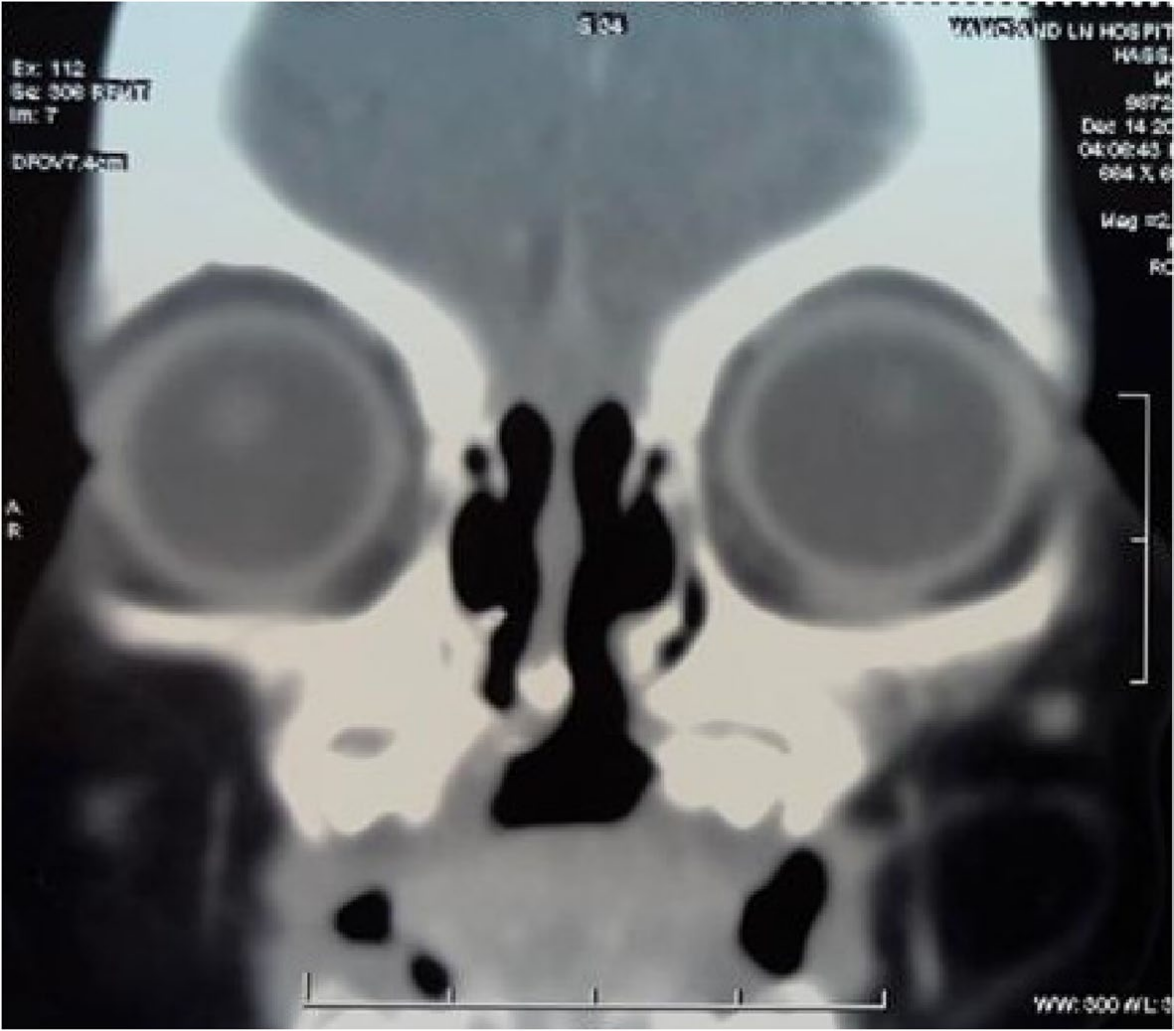
CECT CT face coronal cuts showing the midline palatal defect

**Fig. 3:**
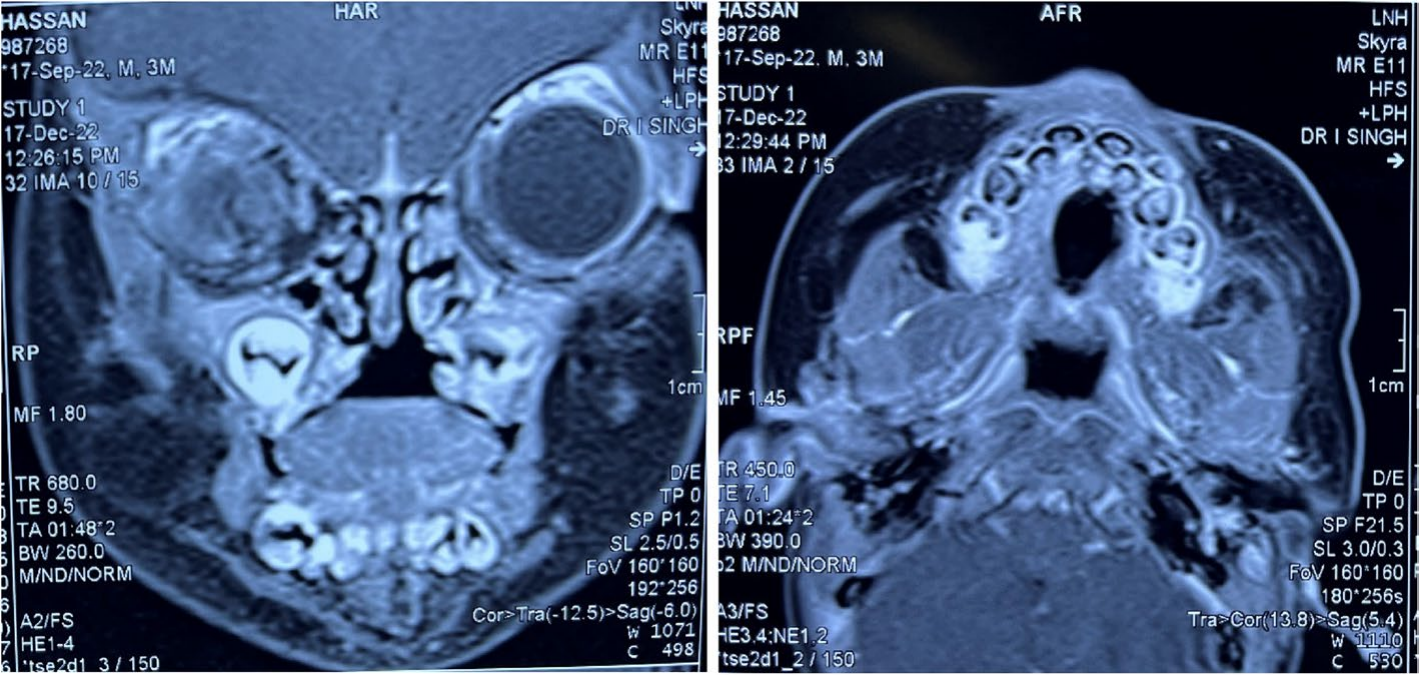
MRI face coronal and axial cuts showing the palatal defect

**Fig. 4:**
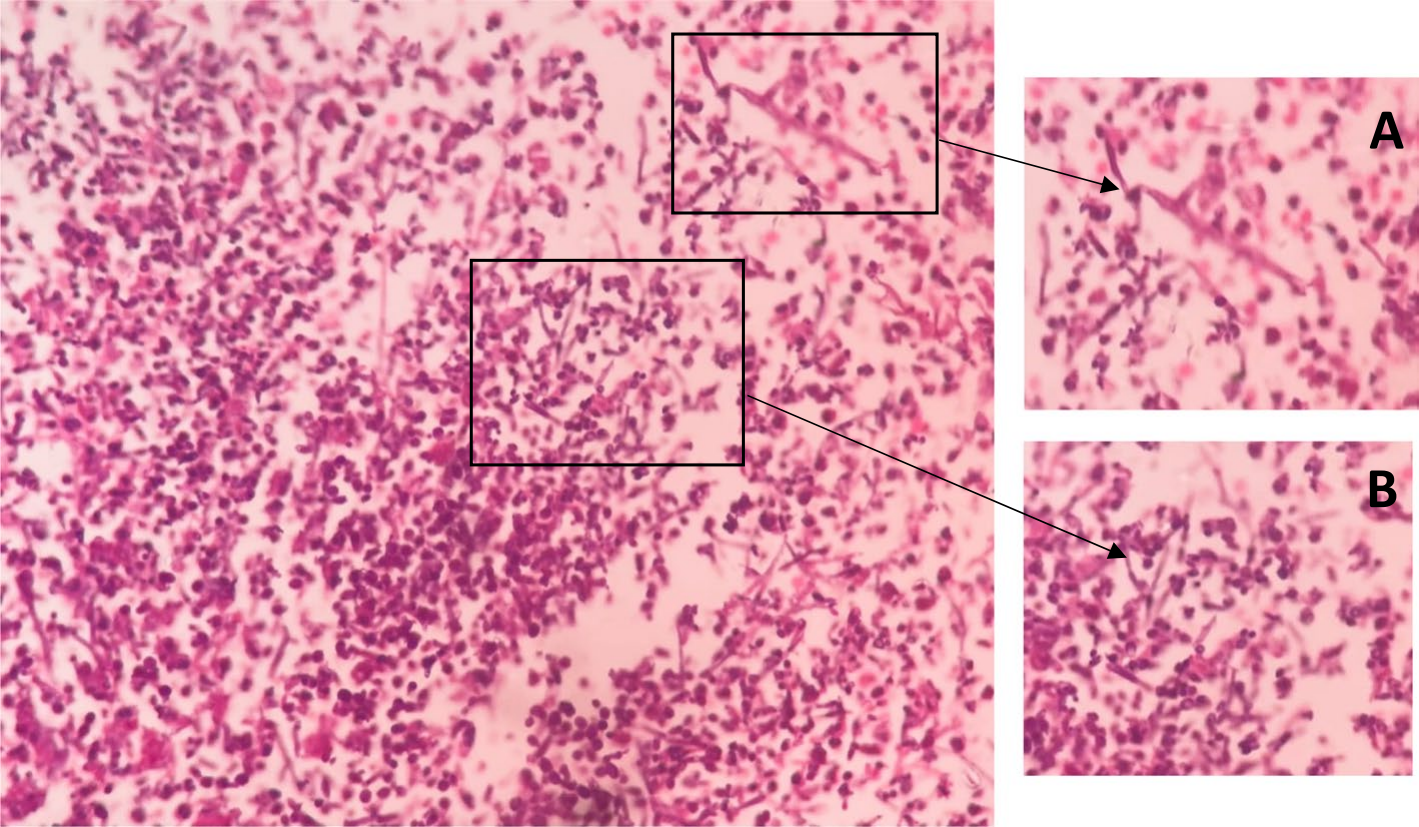
Histopathology showing **A** broad aseptate hyphae with an obtuse branching pattern and **B** septate hyphae with an acute angle branching pattern

A 3-month-old male child presented to the outpatient setting with a complaint of palatal defect for 2 months. A history of black-colored crusting around the defect was present. A history of nasal regurgitation of milk was also present. The patient was apparently well 2 months back when he developed a fever which was insidious in onset, continuous, and not associated with chills and rigor. It was associated with a productive cough. The patient was taken to a private hospital and was diagnosed with pneumonia. The patient did not undergo any COVID testing at the time of his presentation in a private hospital. There was no history of any steroid intake. Following 1.5 months later, the patient developed palatal ulceration which progressed and developed into a defect leading to nasal regurgitation of milk from the nose subsequently landing in our outpatient setting for treatment. The patient had a history of full-term normal vaginal delivery at the hospital, cried immediately after birth, no history of any post-birth complications, and fully vaccinated up to age. No other significant history was noted. Maternal history was taken, and no pre-natal, natal, or post-natal complications were noted. On general physical examination, PR was 123/min, RR 36/min, and SpO_2_ 98% on room air. On oral cavity examination, a palatal defect was found which was nearly 1.5 × 1 cm involving the midpart of the hard palate more toward the right side. It was also extending 0.5 cm posterior to the hard and soft palate junction over the soft palate. The margins of the defect were covered with brownish-dried crust and yellow discharge. Part of the right inferior turbinate and septum could be visualized through the defect. The mucosa of the nasal cavity appeared healthy (Fig. 1). Patient underwent CECT face and neck which showed heterogenous enhancing circumferential mucosal thickening in the developed part of the bilateral maxillary sinuses and ethmoid air cells with associated obliteration of bilateral osteomeatal complex. There was evidence of a large bony defect in the hard palate in the midline measuring nearly 1.2 × 1.0 cm (AP × Tr) leading to a fistulous communication between the oral and nasal cavities (Fig. 2). The patient also have MRI paranasal sinus, orbit, and brain which revealed heterogeneously enhancing mucosal thickening in the developed part of the bilateral maxillary sinus and ethmoid air cells with associated obliteration of bilateral osteomeatal units and large bony defect in the hard palate in the midline with resultant oro-nasal fistula (Fig. 3). Biopsy was taken from the palatal defect which was suggestive of fungal hyphae with broad aseptate, obtuse branching pointing toward mucormycosis; however, few fungal hyphae with septate, acute angle branching suggestive of aspergillosis were also noted on histopathology (Fig. 4). After confirmation on biopsy, patient was started with injection liposomal amphotericin B 12 mg IV daily. He was further planned for surgical debridement and taken up for surgery, intraoperatively margins of the palatal defect were freshened, bone of the hard palate was found to be necrotic which was removed until fresh bleeding was encountered. The sample was sent for histopathological examination. Cross check for immunity profile was done; there was no derangement as per the test conducted.

## Discussion

Mucormycosis and aspergillosis are two opportunistic fungal infections, caused by classes of Zygomycetes and Aspergilli. Zygomycetes have a worldwide distribution, and they are filamentous fungi. They can result in infections of the skin, respiratory tract, lungs, gastrointestinal tract, and rhino-cerebral region. The pathophysiological characteristic of mucorales infection is angio-invasion along with tissue necrosis [1]. Aspergillosis is one of the most rapidly progressing and lethal forms of fungal infection. The number of reported cases has increased threefold in the last years of all the fungal infections, and the related mortality reached up to 85% in the pediatric population, half of the patients succumbed to death within 29 days from the date of diagnosis [2]. Aspergillus damages the tissue from invasion or from the inflammatory cells that are recruited to sites of infection [3, 4]. Primary immunodeficiencies, long-term corticosteroid and/or antibiotic use, protracted neutropenia, AIDS, uncontrolled diabetes mellitus, and malnutrition are high-risk factors for invasive fungal infections. Patients who received bone marrow transplants are particularly vulnerable. In the literature, the prevalence of mucormycosis in such cases ranges from 5 to 14% [5]. Mucormycosis and aspergillosis can both exhibit non-specific, comparable symptoms in the rhino-cerebral and oral-facial region which are primarily fever (71.4%) and rhinorrhea (57.1%) while it can also present with other symptoms like headache, ocular pain, facial edema, and vision anomalies [6]. Primary treatment of mucormycosis is surgical debridement of necrotic area and antifungals like amphotericin. In a study conducted by Dabrits et al. in the pediatric age group out of 12 suspected cases, 9 patients were confirmed for mucormycosis on histopathology. All were started on lipid formulation of liposomal amphotericin B; however, 4 underwent surgical debridement due to soft tissue invasion. One patient undergoing debridement died, other 3 survived while those receiving only medical management, 3 patients died, 1 lost follow-up, and 1 patient was cured. They concluded that for non-soft tissue infection mortality was 88% with medical management [7].

**Fig. 5:**
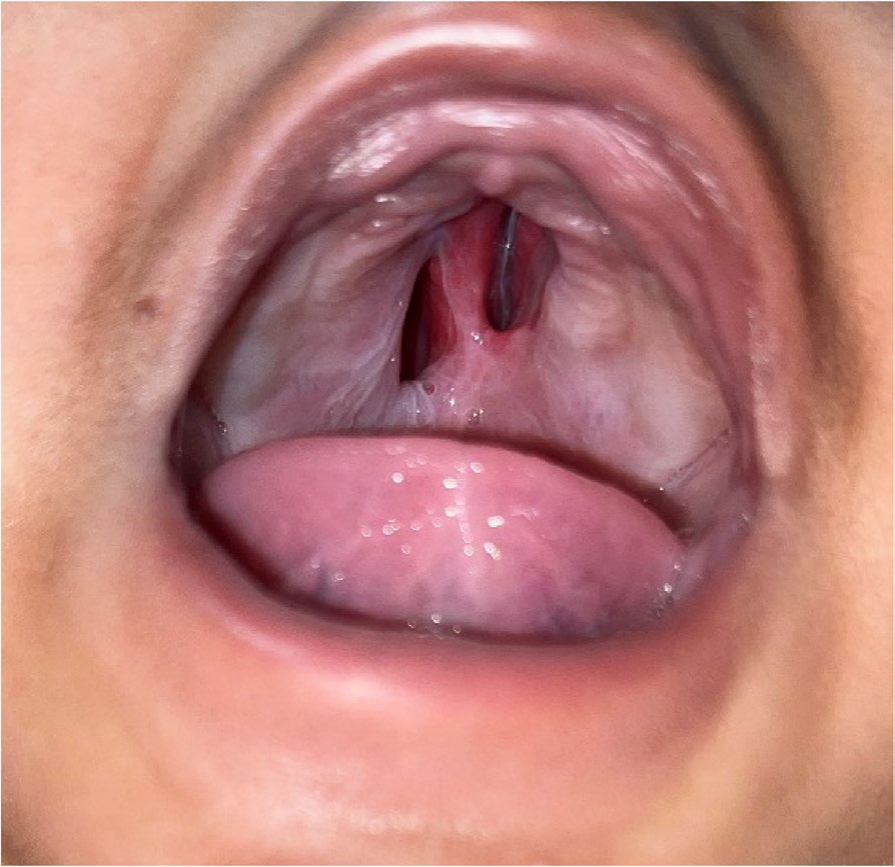
Midline palatal defect post-debridement and medical treatment with healthy margins

In the present study, the patient was diagnosed with pneumonia 1.5 months prior to the development of palatal ulceration; however, test for COVID-19 was not done in this case at the time of diagnosis of pneumonia in a private hospital leading to uncertainty regarding the exclusion of this virus as the cause of pneumonia which in reality can further deteriorate the immunity making patient susceptible to other disease. After the confirmatory diagnosis of mucormycosis and aspergillosis, the child was started on intravenous liposomal amphotericin B as per the recommended dosage of 5 mg/kg/day iv once daily [8]. Voriconazole was not started in the patient; as per a study conducted by Gebremariam et al., it caused hypervirulent Rhizopus and Mucor strains in mice, increasing the lung fungal loads and shortening the lifespans of mice [9]. Our patient underwent both medical and surgical management for disease clearance. Post-debridement patient received a total cumulative dose of 900 mg/dl liposomal amphotericin and was discharged. The patient is currently on follow-up, and the palatal defect is also well-healed with healthy margins (Fig. 5). His parents are well satisfied with the treatment. On reviewing the literature, we could only be able to find four cases of palatal mucormycosis in an infantile age group and none was describing coexisting aspergillosis and mucormycosis in this age group which is described in this case report, and hence, it makes a significant impact in the literature and poses great contribution in the management of such coexisting cases (Table 1).

**Table 1:** Palatal mucormycosis described in the literature in infants

Authors (year)	Age/sex	Presenting complaints	Comorbidity	Treatment	Follow-up
Coetzee et al. [10] (1974)	5 months	Left cheek, eye, eyelid swelling, hard palate sore	History of gastroenteritis treated 2 weeks prior	Debridement + amphotericin B	Recovered
Srivastava et al. [11] (2015)	2 months	Palatal discharge, foul odor, difficulty feeding	History of pneumonia 1 month prior	Debridement + oral voriconazole	Recovered
Singh et al. [12] (2020)	3 months	Failure to accept oral feed, nasal regurgitation of food	Not any	Liposomal amphotericin B	Recovered
Patil et al. [13] (2023)	4 months	Fever, seizure	Ornithine transcarbamylase defect	High-grade antibiotics (meropenam + vancomycin + colistin) and antifungal (not mentioned)	Expired
Present study	3 months	Fever, nasal regurgitation of food	History of pneumonia 1.5 months prior	Debridement + liposomal amphotericin	On follow-up

## Conclusion

Mucormycosis is an infectious fungal disease which is fairly common in adults as compared to the pediatric age group; however, this does not exclude the possibility of this disease in the pediatric age group. These patients can present with palatal ulceration, defect with difficulty feeding, and failure to thrive. In such cases, mucormycosis should be kept in as a differential diagnosis along with other autoimmune and granulomatous conditions. Radiological and histopathological examination is further to be planned, and after confirmation and excluding renal compromise, the patient must be started on liposomal amphotericin B and necrotic foci to be removed via debridement.

## Data Availability

All data generated or analyzed during this study are included in this published article.
